# Role of adenosine in oligodendrocyte precursor maturation

**DOI:** 10.3389/fncel.2015.00155

**Published:** 2015-04-24

**Authors:** Elisabetta Coppi, Lucrezia Cellai, Giovanna Maraula, Ilaria Dettori, Alessia Melani, Anna Maria Pugliese, Felicita Pedata

**Affiliations:** ^1^Department of Health Sciences, University of FlorenceFlorence, Italy; ^2^Department NEUROFARBA, Division of Pharmacology and Toxicology, University of FlorenceFlorence, Italy

**Keywords:** oligodendrocyte progenitor cells, adenosine A_1_ receptors, adenosine A_2A_ receptors, cell differentiation, outward K^+^ currents

## Abstract

Differentiation and maturation of oligodendroglial cells are postnatal processes that involve specific morphological changes correlated with the expression of stage-specific surface antigens and functional voltage-gated ion channels. A small fraction of oligodendrocyte progenitor cells (OPCs) generated during development are maintained in an immature and slowly proliferative or quiescent state in the adult central nervous system (CNS) representing an endogenous reservoir of immature cells. Adenosine receptors are expressed by OPCs and a key role of adenosine in oligodendrocyte maturation has been recently recognized. As evaluated on OPC cultures, adenosine, by stimulating A_1_ receptors, promotes oligodendrocyte maturation and inhibits their proliferation; on the contrary, by stimulating A_2A_ receptors, it inhibits oligodendrocyte maturation. A_1_ and A_2A_ receptor-mediated effects are related to opposite modifications of outward delayed rectifying membrane K^+^ currents (I_K_) that are involved in the regulation of oligodendrocyte differentiation. Brain A_1_ and A_2A_ receptors might represent new molecular targets for drugs useful in demyelinating pathologies, such as multiple sclerosis (MS), stroke and brain trauma.

## Oligodendrocyte Differentiation During Embryonic Development

Oligodendrocytes are neuroglial cells responsible, within the central nervous system (CNS), for myelin sheath formation that provides an electric isolation of axons and accelerates the transmission of electric signals. In order to become able to produce myelin, oligodendroglial cells progress through a series of highly regulated steps of differentiation from OPCs to mature oligodendrocytes (OLGs; Barateiro and Fernandes, 2014). OPCs are generated during embryonic development in restricted areas, such as the subventricular zone (SVZ) and present an amazing migratory ability that allow them to spread and populate brain and spinal cord (El Waly et al., 2014). They are the last cells to be generated after neurons and astrocytes. Their differentiation and maturation are postnatal processes. At the second postnatal day, rodents (rat and mouse) present mainly pre-oligodendrocytes and myelination of the CNS starts only at the seventh postnatal day (Dean et al., 2011). During their maturation, oligodendroglial cells lose their ability to migrate and proliferate (Barateiro and Fernandes, 2014) and acquire an elaborate morphology with many branched processes that wrap around axons and form membrane sheaths of myelin, typical of mature OLGs (de Castro and Bribián, 2005; Table 1).

**Table 1:** **Antigenic pattern typical of the different steps of oligodendrocyte differentiation**.

Oligodendrocyte progenitor cell: OPC	Pre-oligodendrocyte: pre-OLG	Immature OLG	Mature OLG
Nestin^+^	PDGFRα^+^	O4^+^	RIP^+^
PDGFRα^+^	A2B5^+^	RIP^+^	GalC^+^
A2B5^+^	NG2/AN2^+^	GalC^+^	CNP^+^
NG2/AN2^+^	GD3^+^	CNP^+^	MBP^+^
GD3^+^	PLP DM20^+^		PLP^+^
PLP DM20^+^	O4^+^		MAG^+^
Olig2^+^	CNP^+^

Oligodendrocyte maturation involves a sequence of distinct phases that can be identified by the expression of stage-specific surface antigens (Table 1) and by morphological changes (Sommer and Schachner, 1981; Raff et al., 1983; Goldman et al., 1984; Levi et al., 1986; Gard and Pfeiffer, 1990; Warrington and Pfeiffer, 1992; Gallo and Armstrong, 1995; Jung et al., 1996). These characteristics allow for a classification into four stages of differentiation: OPC, pre-oligodendrocyte (pre-OLG), immature oligodendrocyte and mature myelinating oligodendrocyte (OLG; Szuchet et al., 2011; Barateiro and Fernandes, 2014). The initial stage of maturation, as seen at 1–2 days in culture, presents a bipolar (or tripolar) morphology, typical of OPCs (Coppi et al., 2013a). Several are the markers of precocious maturation stages, such as platelet-derived growth factor receptor α (PDGFRα), chondroitin sulfate proteoglycan nerve-glial antigen 2 (NG2) or the transcription factor Olig2 (Pringle et al., 1992; Nishiyama et al., 1996; Ligon et al., 2006). When OPCs start to differentiate in pre-OLGs, they are characterized by emerging secondary ramifications and by the expression of different antigens such as O4 (Sommer and Schachner, 1981). Then they acquire the typical phenotype of immature OLGs characterized by a complex multipolar morphology (Back et al., 2001). At this stage, the expression of markers typical of intermediate steps of maturation, such as O4, persists while markers of the earlier stage such as NG2 and PDGFRα are down regulated (Yu et al., 1994). Finally, when OLGs reach the mature myelinating stage, they acquire highly ramified morphology and immunoreactivity for myelin specific structural proteins such as MAG (myelin associated glycoprotein) and MBP (myelin basic protein) (Scolding et al., 1989; Zhang, 2001). OLGs synthesize large amounts of myelin, giving rise to multilamellar myelin sheath that wrap and isolate neuronal axons.

During their maturation, oligodendrocyte lineage cells display different functional voltage-gated ion channels (Sontheimer et al., 1989) including both inward and outward rectifying K^+^ channels (Sontheimer and Kettenmann, 1988; Williamson et al., 1997), Na^+^ channels (Barres et al., 1990; Berger et al., 1992), and different subtype of Ca^2+^ channels (Verkhratsky et al., 1990; Berger et al., 1992). OPCs (NG2^+^) show outward membrane currents whose main component is represented by delayed rectifying K^+^ currents (I_K_) (Gallo et al., 1996) characterized by low time- and voltage-dependent inactivation and by a threshold of activation at about −40 mV. On the contrary, the transient outward current (I_A_), another K^+^ conductance typical of undifferentiated OPCs, presents a rapid time-dependent inactivation (approximately 50 ms) and a voltage-dependent inactivation at potentials above −40 mV (Gallo et al., 1996). At this immature stage a subpopulation (about 60%) of OPCs also possess an inward, tetrodotoxin-sensitive, Na^+^ current (I_Na_) characterized by a rapid time-dependent inactivation (less than 1 ms) and by a peak of amplitude evoked at about −10 mV (Káradóttir et al., 2008). I_Na_ is never observed in mature oligodendroglial stages (Coppi et al., 2013a).

During development, membrane outward K^+^ conductances (both I_K_ and I_A_) undergo a strong down regulation up to almost completely disappear in mature OLGs (Sontheimer et al., 1989; Barres et al., 1990; Gallo et al., 1996; Attali et al., 1997; Coppi et al., 2013a).

In parallel with outward K^+^ current downregulation, there is a gradual increase in the expression of inwardly rectifying K^+^ currents (K_ir_), activated at potentials more negative than membrane resting potential (Vm, about −70 mV). K_ir_ currents are the main conductance represented in mature OLGs (Knutson et al., 1997).

Among the mentioned currents, voltage-gated K^+^ currents, such as I_K_, I_A_ and K_ir_, are involved in the regulation of oligodendrocyte differentiation and thus of myelin formation (Sontheimer et al., 1989; Gallo et al., 1996). In addition, the expression of I_K_ currents is linked to cell cycle regulation and hence to proliferative capacity of OPCs (Chittajallu et al., 2005) because of the following: (1) a down regulation of I_K_ occurs as oligodendrocyte lineage cells mature (Sontheimer et al., 1989; Barres et al., 1990); (2) proliferative OPCs express larger I_K_ currents than cell cycle-arrested OPCs (Knutson et al., 1997; Chittajallu et al., 2002); and (3) pharmacological block of I_K_ induced by tetra-ethyl-ammonium (TEA) in cells belonging to the oligodendrocyte lineage is sufficient to delay their proliferation and differentiation (Gallo et al., 1996; Chittajallu et al., 2002). Hence, treatments aimed at modulating these currents may affect oligodendrocyte proliferation and differentiation.

## Adult Oligodendrocyte Progenitor Cells

It has been demonstrated that a small fraction of OPCs generated during development are maintained in an immature and slowly proliferative or quiescent state in the adult CNS (Dawson et al., 2003) where they are called “adult OPCs”. Adult OPCs are present in all brain structures where they represent the 2–9% of the total cellular population of the CNS (Dawson et al., 2003) thus being the largest population of proliferating cells within the CNS (Horner et al., 2000). Adult OPCs persists stable in the adult CNS (Rivers et al., 2008) where they represent an endogenous reservoir of immature cells (de Castro and Bribián, 2005), constantly produced by neural stem cells located in the SVZ (Menn et al., 2006). Current evidence suggests that adult OPCs express the same markers (e.g., NG2 or PDGFRα) and appear morphologically similar to their developmental counterpart (Franklin and Ffrench-Constant, 2008).

Very little is known about factors that control adult oligodendrogenesis. Upon opportune physiological (e.g., voluntary physical exercise; Simon et al., 2011) or pathological (e.g., injury, inflammation, demyelination) stimuli, adult OPCs are able to react with increased proliferation and subsequent differentiation in mature OLGs (Simon et al., 2011), thus representing the primary source of myelinating cells in the CNS (Nishiyama et al., 1999; Windrem et al., 2004).

Self-renewal and multipotency features of adult OPCs have been reported. Thanks to their self-renewal features, adult OPCs continue to proliferate throughout life span (Young et al., 2013) and to differentiate into mature OLGs, ensuring myelin integrity. Under specific conditions, adult OPCs give rise to neurons, astrocytes (Nishiyama et al., 2009) and Schwann cells (Zawadzka et al., 2010). However, multipotency of adult OPCs is still to be confirmed since remains controversial due to some discrepancy between *in vitro* and *in vivo* data (Crawford et al., 2014).

Adult OPCs have been shown to contact the axonal membrane (Butt et al., 1999) and the synaptic terminals (Ong and Levine, 1999). This raises the question of whether adult OPCs may be capable to impact or to react to neuronal activity (Butt et al., 2002, 2005; Nishiyama et al., 2002). Concerning this topic a recent study demonstrated the positive impact of neuronal activity on myelination in the adult brain (Gibson et al., 2014).

## Adenosine and Oligodendrocyte Maturation

It is known that purines, in addition to their functions as neurotransmitters and neuromodulators, can also act as growth and trophic factors, thus influencing the development of neuronal (Mishra et al., 2006; Migita et al., 2008) and glial (Stevens and Fields, 2000; Stevens et al., 2002) cells.

All adenosine receptor subtypes (A_1_, A_2A_, A_2B_, A_3_) are expressed on different cell types within the CNS including oligodendrocytes, likely being able to modulate cell-to-cell communication between neurons and glial cells (Othman et al., 2003; Agresti et al., 2005).

The expression by oligodendrocytes of the equilibrative nucleoside transporters ENT1 and ENT2, as well as adenosine degrading enzymes such as adenosine deaminase and adenosine kinase has been demonstrated (González-Fernández et al., 2014). All adenosine receptor subtypes are also expressed by OPCs (Stevens et al., 2002; Fredholm et al., 2011) and a key role of adenosine in oligodendrocyte maturation has been recognized (Burnstock et al., 2011). In particular it was demonstrated that adenosine can affect numerous OPC processes such as migration, proliferation and maturation (Stevens and Fields, 2000; Stevens et al., 2002; Coppi et al., 2013a).

### Adenosine A_1_ Receptor-Mediated Effects on Oligodendrogenesis

Treatment of cultured OPCs with adenosine exerts a concentration-dependent reduction of their proliferation in the presence of the mitogen PDGF and promotes cell differentiation towards pre-myelinating oligodendrocytes, an effect that is mediated by A_1_ receptor (Stevens et al., 2002). A chronic adenosine treatment in co-cultures of OPCs with dorsal root ganglion neurons also promotes myelination as shown by the rise of MBP^+^ cells after 14 days (Stevens et al., 2002). Of note, the percentage of myelinating MBP^+^ OLGs was lower in co-cultures treated with the adenosine receptor antagonist, suggesting that endogenous sources of adenosine are sufficient to promote OPC differentiation (Stevens et al., 2002). In addition, the activation of A_1_ receptor has been reported to induce OPC migration (Othman et al., 2003).

On these basis, it was proposed that activation of A_1_ receptors on OPCs by extracellular adenosine allows for the beginning of the myelination process possibly offering new approaches for the treatment of demyelinating diseases in the CNS, such as MS (Stevens et al., 2002).

Such an effect, however, is different from what has been described in neonatal rats. Neonatal rats treated with A_1_ receptor agonists showed a marked reduction in white and gray matter volume and ventriculomegaly (Turner et al., 2002) with reduced expression of MBP similarly to what observed in neonatal rats reared in hypoxia (Ment et al., 1998). Ventriculomegaly was also observed in mice lacking the enzyme adenosine deaminase which degrades adenosine (Turner et al., 2003). Moreover, hypoxia-induced periventricular white matter injury (PWMI, a form of brain injury sustained by preterm infants) was prevented in mice lacking A_1_ receptor (Turner et al., 2003). These data support the notion that adenosine, acting on A_1_ receptor, mediates hypoxia-induced brain injury and ventriculomegaly during early postnatal development (Turner et al., 2003). Such effect could be attributed to the fact that adenosine released under hypoxia and acting on A_1_ receptors leads to premature differentiation and reduced proliferation of oligodendroglia precursors. In fact, studies of OPCs and pre-OLGs in hypoxic conditions revealed a reduced proliferation and an accelerated maturation, as demonstrated by the increased expression of the cell cycle regulatory proteins p27 (Kip1) and phospho-cdc2 (Akundi and Rivkees, 2009). This series of events would lead to a reduced number of OLGs available for myelination, thus contributing to PWMI (see in Rivkees and Wendler, 2011). Thus, strategies aimed at stimulating OPC proliferation in neonatal hypoxia/ischemia may be of value to prevent PWMI.

### Adenosine A_2A_ Receptor-Mediated Effects on Oligodendrogenesis

**Figure 1:**
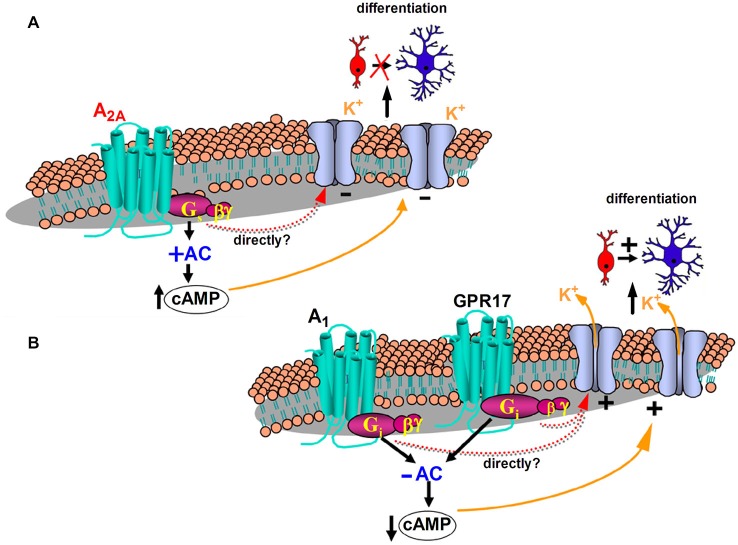
**Proposed mechanisms underlying K^+^ channel modulation and differentiation of oligodendrocyte progenitor cells (OPCs: red cells) into mature oligodendrocytes (blue cells) after purinergic receptor activation**. **(A)** Stimulation of the Gs coupled adenosine A_2A_ receptor subtype increases adenyl cyclase (AC) activity and intracellular cyclic adenosine monophosphate (cAMP), decreases the amplitude of I_K_ currents recorded from cultured OPCs and inhibits OPC differentiation (Coppi et al., 2013b). **(B)** The Gi-coupled adenosine A_1_ receptor decreases AC activity and intracellular cAMP, increases the amplitude of I_K_ currents recorded from cultured OPCs (Coppi et al., 2013b) and promotes OPCs to more mature stages of differentiation (Stevens et al., 2002). Increased amplitude of I_K_ currents is obtained also by selective stimulation of GPR17 (Coppi et al., 2013a), a recently deorphanized Gi-coupled P2Y-like receptor, which decreases AC activity and intracellular cAMP and stimulates OPC maturation (Lecca et al., 2008; Fumagalli et al., 2011). Modulation of I_K_ currents may be directly driven by G-protein subunit/s or indirectly through modulation of intracellular cAMP levels.

The first functional characterization of adenosine A_2A_ receptors in OPCs has been recently reported (Coppi et al., 2013b). It was demonstrated that the selective A_2A_ receptor agonist CGS21680 inhibits I_K_ currents in cultured OPCs with an EC_50_ in the low nanomolar range (which is in line with values reported in the literature: see Jarvis et al., 1989; Fredholm et al., 2011). Furthermore, CGS21680 inhibits *in vitro* OPC differentiation since it increases the percentage of NG2^+^ immature OPCs and reduces O4^+^ pre-OLGs and MAG^+^ mature OLGs, without affecting neither cell viability nor cell proliferation (Coppi et al., 2013b). These effects are completely prevented in the presence of the selective A_2A_ receptor antagonist SCH58261. TEA, at concentrations that block I_K_ but not I_A_ currents, mimics and occludes the effect of the A_2A_ agonist on membrane currents, confirming a selective modulation by this purinergic receptor subtype of I_K_ currents (Coppi et al., 2013b). Similar effects mediated by A_2A_ receptors on outward rectifying K^+^ channels have already been described in other cell types (Xu and Enyeart, 1999; Duffy et al., 2007), with an involvement of either intracellular cAMP rise or a direct action of the Gs protein coupled to A_2A_ receptors being hypothesized. Of note, we have demonstrated that the activation of GPR17, a recently deorphanized Gi-coupled P2Y-like receptor, which stimulates OPC differentiation (Lecca et al., 2008; Fumagalli et al., 2011), elicits opposite effects in comparison to the Gs coupled A_2A_ subtype, thus increasing the amplitude of I_K_ currents recorded from cultured OPCs (Coppi et al., 2013a). Increased amplitude of I_K_ currents is obtained also by selective stimulation of adenosine, Gi-coupled, A_1_ receptors (unpublished observation mentioned in Coppi et al. (2013b)). Taken together, these data suggest that the intracellular signaling pathway leading to cAMP decrease in OPC cultures are positively coupled to I_K_ currents and cell differentiation (Figure 1B).

In keeping with data demonstrating that the inhibition of I_K_ currents impairs proliferation and maturation of cultured OPCs (Gallo et al., 1996; Attali et al., 1997; Ghiani et al., 1999; Chittajallu et al., 2002; Vautier et al., 2004) and blocks myelin deposition in embryonic spinal cord (Shrager and Novakovic, 1995), it appears that A_2A_ receptor stimulation inhibits OPC differentiation by reducing I_K_ currents (Figure 1A). In line with this assumption, it has been observed that adenosine A_1_ receptors, which enhances I_K_ currents in OPCs, exert a pro-differentiating effect in oligodendrocyte cultures (Stevens et al., 2002; Figure 1B). However, we cannot exclude that other intracellular pathways, in addition to I_K_ current block, contribute to the A_2A_ receptor-mediated inhibition of OPC differentiation. In OPCs, the tyrosine kinase fibroblast growth factor receptor (FGF) is also expressed and its activation promotes cell proliferation and inhibits the expression of myelin components (Besnard et al., 1989). On the contrary, the simultaneous activation of both A_2A_ and FGF receptors, by robust activation of the mitogen activated protein kinase (MAPK/ERK) pathway, brings to differentiation and neurite extension of PC12 cells (Flajolet et al., 2008). It is likely that also in OPCs a cross talk between the two receptors regulates cell maturation.

Upregulation of A_2A_ receptor has been observed in cerebral white matter of patients with secondary progressive MS: higher density of brain A_2A_ receptor appeared correlated with higher disability scale scores in MS patients (Rissanen et al., 2013). These data were interpreted as A_2A_ receptor upregulation on brain cells is associated with the disease progression. In agreement, in a mouse model of experimental autoimmune encephalomyelitis (EAE: an animal model for MS), adenosine A_2A_ antagonists protected from disease development (Mills et al., 2012), suggesting that activation of adenosine A_2A_ receptors on neuronal and glial cells is responsible for EAE development in mice. Moreover in a rat model of focal brain ischemia (the middle cerebral artery occlusion: MCAO model), adenosine A_2A_ receptor antagonists systemically administered after ischemia prevented the activation of JNK mitogen activated kinase (Melani et al., 2009) that, by activating caspase 3 and the pro-apoptotic regulator DP5 (Yin et al., 2005), is involved in oligodendrocyte cell death (Howe et al., 2004; Jurewicz et al., 2006). Selective A_2A_ adenosine antagonists also prevented the myelin disorganization in the basal nuclei and reduced the expression of Olig2 (Melani et al., 2009), a marker of immature OPCs, poorly expressed by mature OLGs.

In light of these data, it can be postulated that, under demyelinating conditions, the A_2A_ receptor-mediated inhibition of OPC maturation is associated with an increased damage, since the stimulation of this receptor subtype prevents myelin deposition.

Such a role of adenosine A_2A_ receptor might appear in contrast with the observation that genetic ablation of both central and peripheral A_2A_ receptors exacerbates brain damage and neuroinflammation in a mouse model of EAE (Yao et al., 2012). In fact, A_2A_ receptors on peripheral leucocytes are known to reduce adhesion cell factor production and neutrophil activation (Sitkovsky et al., 2004). Thus, it is likely that genetic ablation of adenosine A_2A_ receptor on blood cells is crucial in exacerbating leucocyte infiltration, neuroinflammation and brain damage in a model of chronic inflammation such as EAE (see Pedata et al., 2014). A regulation of neuroinflammation by adenosine receptors might be critical in the modulation of myelin repair mechanisms in different neurodegenerative diseases affecting the CNS.

## Effect of Caffeine on the White Matter Injury in Hypoxic Neonatal Brain

Caffeine is widely used in neonatal medicine in order to stimulate the respiration in premature infants. Apnea of prematurity (AOP) is a significant clinical problem manifested by an unstable respiratory rhythm reflecting the immaturity of respiratory control systems. AOP typically resolves with maturation suggesting that increased myelination of the brainstem is required for disease remission (Mathew, 2011). This raises the question of whether caffeine improves the myelination and therefore the immaturity of respiratory control systems.

In hypoxia-exposed neonatal mice pups treated with caffeine, myelination was enhanced and ventriculomegaly reduced from postnatal days 3 through 12; furthermore, more normally arranged myelinated axon orientation than that observed in hypoxia was reported (Back et al., 2006). Caffeine also increased the percentage of immature OLGs in the brain tissue (Back et al., 2006). Further support for the notion that caffeine is neuroprotective in development is provided by non-randomized studies demonstrating that improved myelination is observed in premature baboons treated with caffeine (Loeliger et al., 2006).

Caffeine is a non-selective antagonist of A_1_ and A_2A_ receptors, thus increased myelination could be ascribed to the inhibition of one or both A_1_ and A_2A_ receptor subtype. Since adenosine A_1_ receptors are causative of hypoxia-induced brain injury and ventriculomegaly during early postnatal development (Turner et al., 2002, 2003), antagonism of adenosine A_1_ receptors is relevant in the action mechanism of caffeine.

## Conclusions

As evaluated directly on cultured OPCs, adenosine acts as a dual modulator of OPC development since, by stimulating A_1_ receptors, it promotes oligodendrocyte maturation and inhibits their proliferation (Stevens et al., 2002) and, on the contrary, by stimulating A_2A_ receptors, it inhibits oligodendrocyte maturation (Coppi et al., 2013b).

Most probably, the effects of adenosine on OPC maturation are of relevance during demyelinating pathologies, such as MS, EAE or ischemia. However, it appears that a premature differentiation and reduced proliferation of OPCs might compromise the process of myelination as in PWMI in preterm infants. The outcome of stimulation of adenosine A_1_ and A_2A_ receptors on myelination depends likely on the timing of their stimulation during the development and on the different demyelinating pathologies.

Identification of critical regulators that inhibit myelination/remyelination could facilitate the development of therapeutic targets for myelin repair in CNS demyelinating diseases. For example, the fact that in MS lesions OPCs are present but fail to differentiate into mature OLGs (Levine and Reynolds, 1999; Chang et al., 2000), suggests that the remyelination process is blocked at a premyelinating stage.

Adenosine A_1_ and A_2A_ receptors (to date no investigations concerning the role of adenosine A_3_ or A_2B_ receptors on OPC maturation have been performed) represent new possible molecular targets for drugs useful in demyelinating pathologies, such as MS, stroke and brain trauma.

## Conflict of Interest Statement

The authors declare that the research was conducted in the absence of any commercial or financial relationships that could be construed as a potential conflict of interest.
